# Gender Professionalism: Redefining Respect in Healthcare and Medical Education

**DOI:** 10.12669/pjms.41.9.12982

**Published:** 2025-09

**Authors:** Wajiha Rizwan, Saima Chaudhry

**Affiliations:** 1 Wajiha Rizwan President, Medical Women’s Association Pakistan, Deputy Director DME, University of Child Health Sciences, Lahore, Pakistan; 2 Saima Chaudhry Director Medical Women’s Foundation Pakistan. Director CHPL, The University of Lahore, Lahore, Pakistan

**Keywords:** Gender Professionalism, Healthcare, Medical Education, Respect

The value of professionalism in healthcare is immeasurable, forming the bedrock of trust, safety and ethical practices across all domains of the field. It is a multidimensional construct that encompasses not only technical competence but also a set of non-cognitive skills, behaviors and ethical principles.^1^ In the healthcare sector, this contract assumes paramount importance, as the well-being of the patients, the functionality of multidisciplinary teams, the advancement of medical science are all based on a framework of unquestionable professionalism. However, this ideal is frequently challenged by complex dynamics of gender, which can implicitly or explicitly compromise the very principles upon which professional conduct is established.^2^ While these principles are universally applicable their implementation challenges are profoundly shaped by specific cultural, social, and institutional contexts.

In Pakistan, a nation characterized by rich cultural heritage and a rapidly evolving healthcare landscape the need for a robust framework for gender professionalism is more critical than ever. Despite the notable surge in female enrollment in medical colleges with some reports indicating that women constitute up to 70% of students, this educational progress is not consistently translating into sustained professional careers.^3^ A significant proportion of female medical graduates either do not enter clinical practice or prematurely withdraw from their profession.^4^ This phenomena of female doctor attrition represents a matter of national concern contributing to a persistent shortage of medical professionals and signifying substantial loss of human capital and investment. The underlying factors for this situation are multifaceted where a central theme is the intersection of a deeply patriarchal societal structure and a healthcare system that has not yet fully adapted to a gender-diverse workforce.^5^

Within a society where traditional gender roles are often rigidly defined, demarcation between personal and professional relationships can become blurred, leading to instances of exploitation and misconduct.^6^ This results in challenges encountered by healthcare professionals in Pakistan beyond issues of work-life balance, rooted in systemic problems of gender-based discrimination, harassment, and the work environment that is often not conducive to their professional growth. One of the most critical manifestations of this challenge is the failure to enforce and respect professional boundaries. Female doctor and nurses frequently encounter harassment not only from patients and their attendants but also from male colleagues and supervisors. This can range from inappropriate verbal remarks and unwanted advances to more severe forms of bullying and physical violence. A study found that 43% of female healthcare workers had experienced some workplace violence, with verbal and psychological forms being the most prevalent.^7^ Gender-based misconduct is not confined to the harassment of female professionals. A systematic review on workplace violence in Pakistan has revealed that physical abuse is most frequently experienced by male healthcare providers. Moreover, male professionals are reported to be victims of sexual harassment and bullying at the hands of senior staff or patients,^8^ an issue that is often underreported due to societal expectations of masculinity and the fear of not being taken seriously. Also, a male healthcare professional interacting with the female patient may need to navigate cultural barriers and misinterpretations that require heightened level of gender sensitivity.

**Fig-1:**
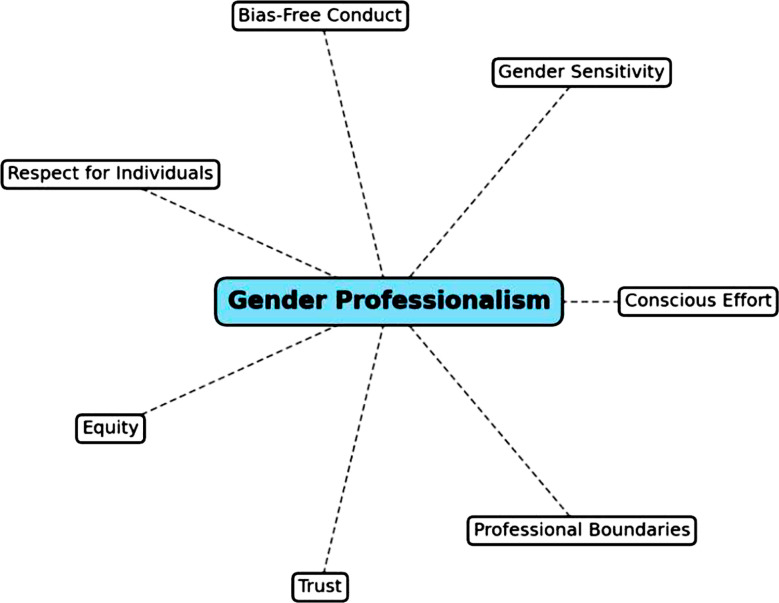
Components of Gender Professionalism

The underreporting of all incidents of these unprofessional behaviors by all genders, driven by fear of stigmatization, professional retaliation, and a lack of confidence in institutional support mechanisms perpetuates the cycle of impunity and risk, highlighting the systematic nature of the issues.^9^ A genuine healthcare system must skillfully navigate these complexities, ensuring authentic professionalism in all contexts, without defaulting to gendered assumptions. Therefore, the need for *Gender Professionalism*, the term coined by the authors to describe *the deliberate and conscious effort to ensure that professional behavior is gender-sensitive, free from bias, and respectful to all individuals, fostering equity and trust in professional relationships*. This vision includes maintaining clear professional boundaries, encouraging respectful interactions, and creating environments where all individuals feel safe and valued (Fig 1).

Addressing this issue requires a holistic and institutional approach that goes beyond mere problem recognition. It necessitates a dedicated institutionally backed effort to cultivate a pervasive culture of gender professionalism. The initial step is mandatory training for all healthcare professionals, from students to senior faculties that must move beyond the simplistic enumeration of rules and must focus on building a profound understanding of gender-sensitive behavior, the critical importance of professional boundaries, and the established mechanisms for reporting and addressing misconduct. The training should lead to integrating Gender Professionalism module into curricula of healthcare institutions and continuing professional development programs. The trained and aware workforce can then be instrumental in developing implementable policies and advocate systemic change to the professional bodies, government health departments, and hospital administrations. Enforceable policies against gender-based discrimination and harassment advocacy in this context becomes a continuous process of not only educating the workforce, but also of influencing the very structures and policies that govern it, thereby ensuring that the principles of gender professionalism are protected and upheld for all.
